# Supplementary material for: The feeding microstructure of male and female mice
Source: PLoS One. 2021 Feb 4;16(2):e0246569. doi: 10.1371/journal.pone.0246569 (PMC7861458; doi:10.1371/journal.pone.0246569)
Supplement: S1 Fig — The study design is shown as a 2wk interval timeline spanning the entire experiment (~35 weeks). As indicated, ten male and female mice were weaned at week 3 of age (p19-20). When mice were not monitored for their feeding behavior, they were housed in groups of 5 mice per cage per sex. When mice were 8-9wk old, they were implanted with unique RFID chips. Blue arrowheads indicate the time where male or female mice housed in groups of 5 animals were randomly swapped between their 2 respective cages. This was done a week before and immediately after monitoring their feeding behavior, which are represented as red rectangles. Before and during the 3 weeks of monitoring their feeding behavior, male or female mice were housed in groups of 10, as indicated by green arrowheads. Purple arrowheads point out the times where body composition was determined. (PDF) [file pone.0246569.s001.pdf]

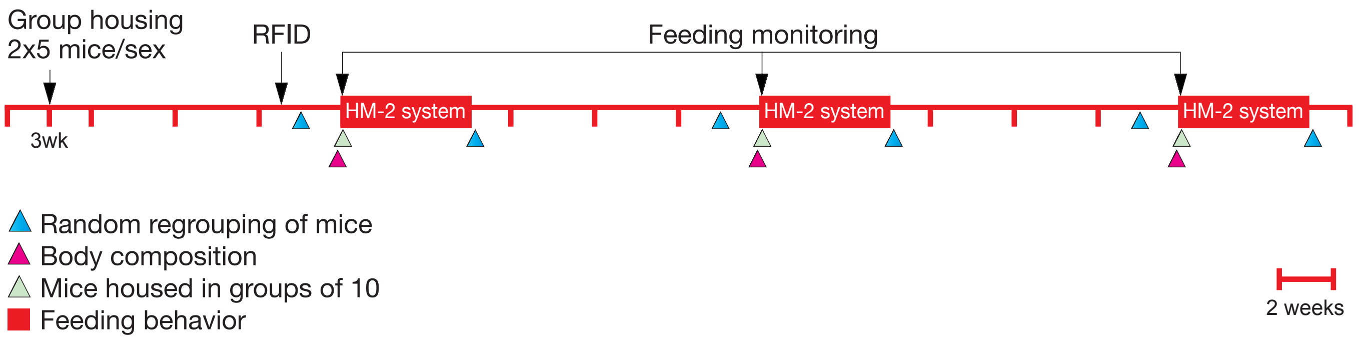


**S1 Fig.** ***Experimental design timeline.*** The study design is shown as a 2wk interval timeline spanning the entire experiment (~35 weeks). As indicated, ten male and female mice were weaned at week 3 of age (p19-20). When mice were not monitored for their feeding behavior, they were housed in groups of 5 mice per cage per sex. When mice were 8-9wk old, they were implanted with unique RFID chips. Blue arrowheads indicate the time where male or female mice housed in groups of 5 animals were randomly swapped between their 2 respective cages. This was done a week before and immediately after monitoring their feeding behavior, which are represented as red rectangles. Before and during the 3 weeks of monitoring their feeding behavior, male or female mice were housed in groups of 10, as indicated by green arrowheads. Purple arrowheads point out the times where body composition was determined.
