# Supplementary material for: The feeding microstructure of male and female mice
Source: PLoS One. 2021 Feb 4;16(2):e0246569. doi: 10.1371/journal.pone.0246569 (PMC7861458; doi:10.1371/journal.pone.0246569)
Supplement: S2 Fig — Shown are the meal size (A, kCal), frequency (B, counts per mouse), duration (C, seconds) and intermeal interval (D, minutes) of group-housed male and female mice at 10w, 20w and 30w of age fed ad libitum a chow diet. Diurnal data was recorded during 14 consecutive days by using two IMI threshold criteria: ≥5min and ≥8min. Results are expressed as the mean ± SEM. (PDF) [file pone.0246569.s002.pdf]

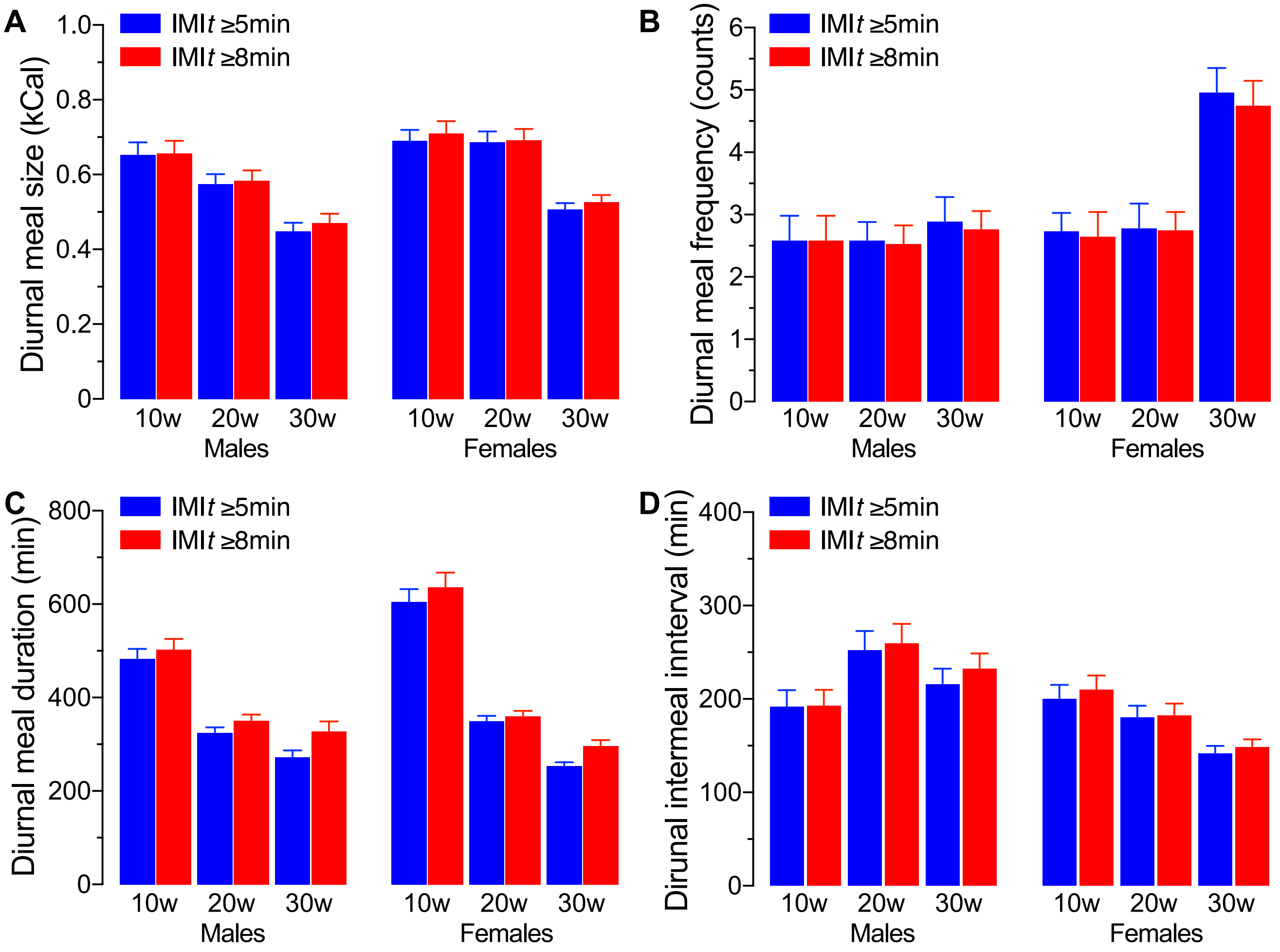

S2 Fig. ***The diurnal feeding microstructure of group-housed mice computed by using IMI ≥5min and IMI ≥8min.*** Shown are the meal size (A, kCal), frequency (B, counts per mouse), duration (C, seconds) and intermeal interval (D, minutes) of group-housed male and female mice at 10w, 20w and 30w of age fed ad libitum a chow diet. Diurnal data was recorded during 14 consecutive days by using two IMI threshold criteria: ≥5min and ≥8min. Results are expressed as the mean ± SEM.
