# Supplementary material for: The feeding microstructure of male and female mice
Source: PLoS One. 2021 Feb 4;16(2):e0246569. doi: 10.1371/journal.pone.0246569 (PMC7861458; doi:10.1371/journal.pone.0246569)
Supplement: S3 Fig — A. Net energy intake (kCal/mouse) of 10w, 20w and 30w old males and females continuously recorded during 14 days while fed ad libitum a chow diet (3.0 kCal/g). Results represent the mean ± SEM (n = 10, *p<0.05 sex; ●p<0.05 vs. 10w old mice). B. Mean nocturnal and diurnal ambulatory activity integrated from data shown in Fig 2D. Results are expressed as the mean ± SEM (n = 10, ●p<0.05 vs. 10w old mice). C-E. Energy intake of 10w (C), 20w (D) and 30w (E) old males and females cumulated over 14 days of ad libitum feeding a chow diet. Dashed lines indicate the mean net daily energy intake (*p<0.05 sex). (PDF) [file pone.0246569.s003.pdf]

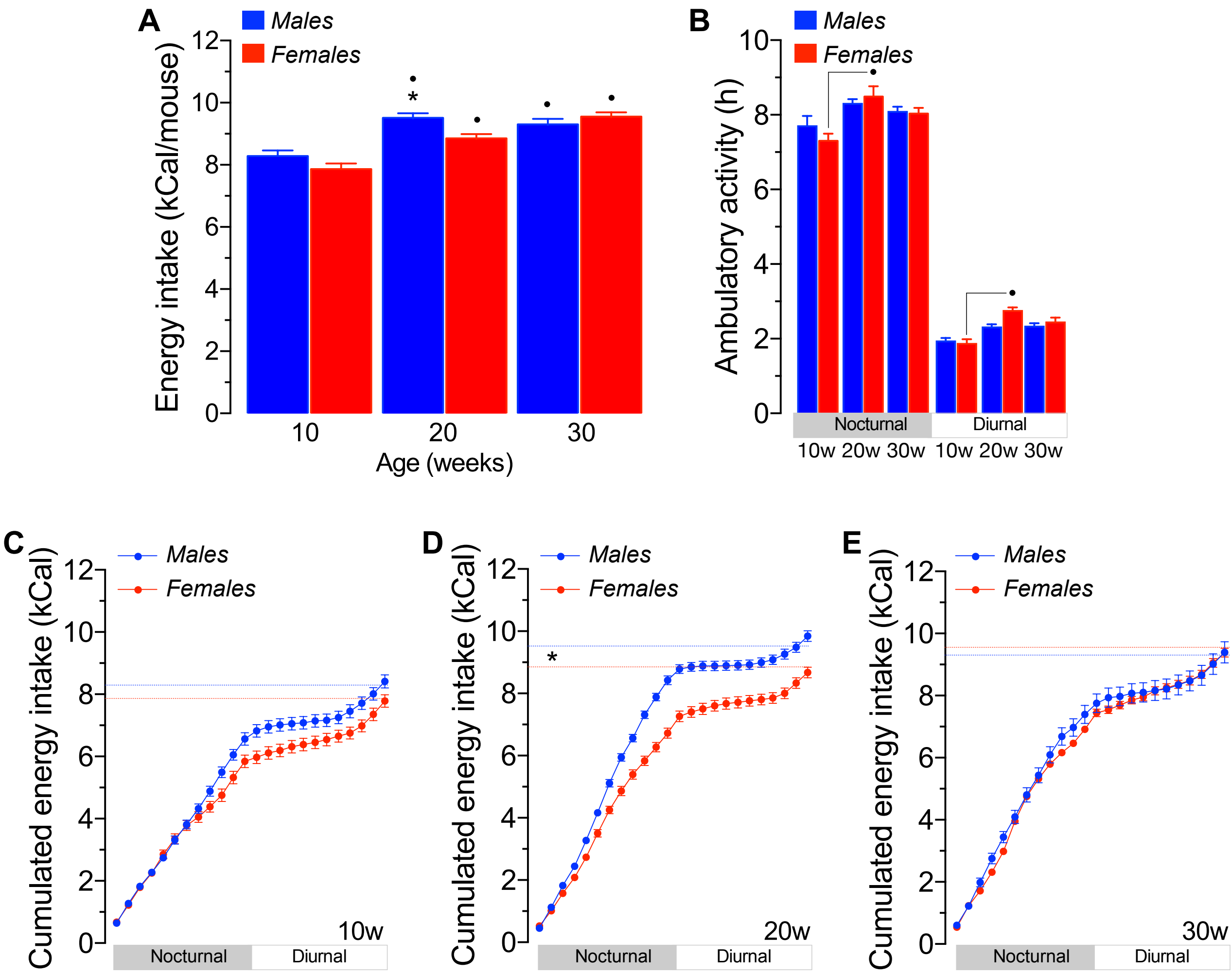

S3 Fig. ***Net and cumulated daily energy intake and ambulatory activity of normal mice housed in groups.*** **A.** Net energy intake (kCal/mouse) of 10w, 20w and 30w old males and females continuously recorded during 14 days while fed *ad libitum* a chow diet (3.0 kCal/g). Results represent the mean ± SEM (*n*=10, **p*<0.05 sex; •*p*<0.05 *vs.* 10w old mice). **B.** Mean nocturnal and diurnal ambulatory activity integrated from data shown in Figure 2D. Results are expressed as the mean ± SEM (*n*=10, •*p*<0.05 *vs.* 10w old mice). **C-E.** Energy intake of 10w (C), 20w (D) and 30w (E) old males and females cumulated over 14 days of *ad libitum* feeding a chow diet. Dashed lines indicate the mean net daily energy intake (**p*<0.05 sex).
