# Supplementary material for: The feeding microstructure of male and female mice
Source: PLoS One. 2021 Feb 4;16(2):e0246569. doi: 10.1371/journal.pone.0246569 (PMC7861458; doi:10.1371/journal.pone.0246569)
Supplement: S4 Fig — A. Shown are the net core body temperature (BT) change in group-housed male and female mice in response to 16hs of fasting (A) and basal BT obtained in mice fed ad libitum (C). Results represent the mean ± SEM (n = 10, *p<0.001 sex; ●p<0.05 vs. 10w old mice). C. Ambulatory activity of 10w, 20w and 30w old male and female during 16hs of fasting. (PDF) [file pone.0246569.s004.pdf]

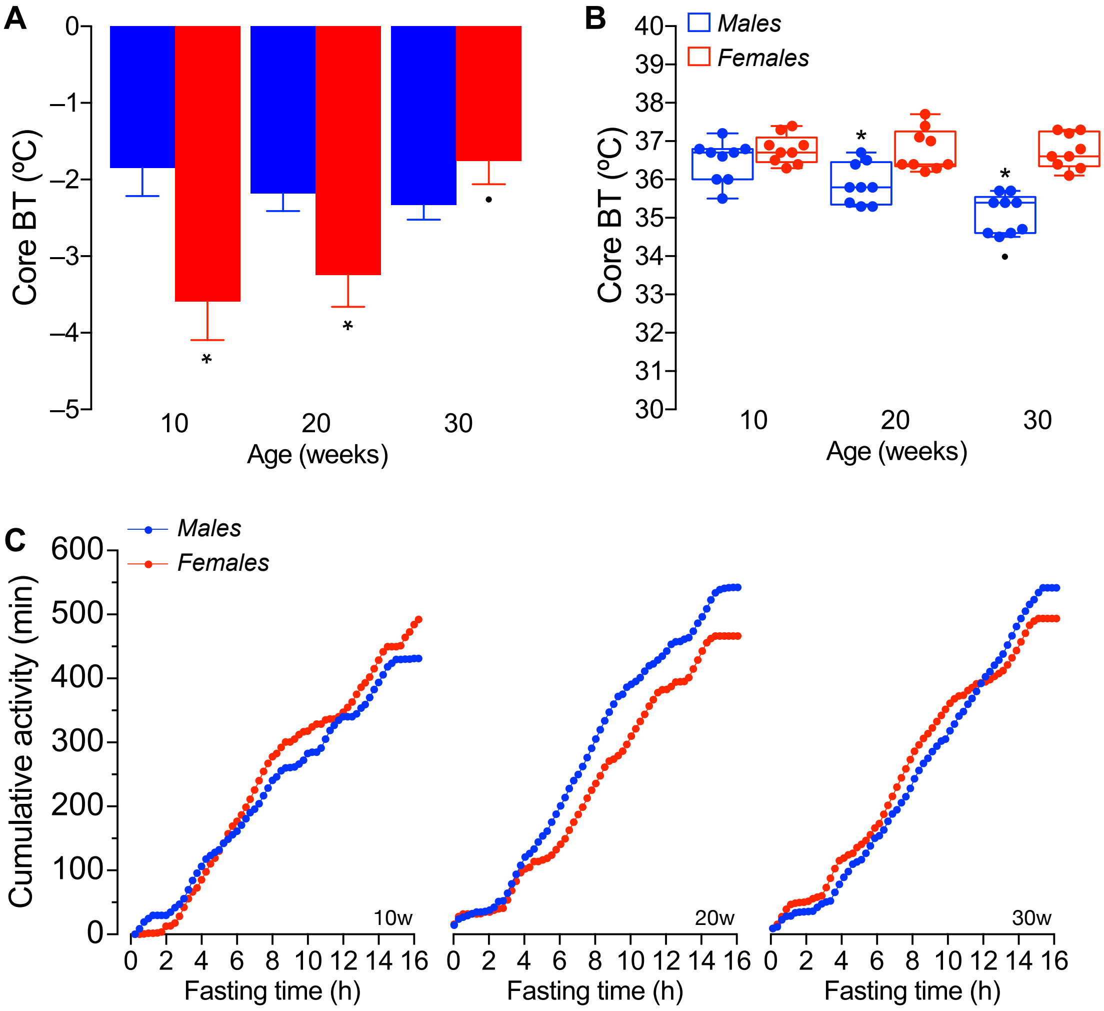


**S4 Fig. *Fasting-induced hypothermia and cumulated ambulatory activity during fasting in normal mice housed in groups.* A.** Shown are the net core body temperature (BT) change in group-housed male and female mice in response to 16hs of fasting (A) and basal BT obtained in mice fed *ad libitum* (C). Results represent the mean ± SEM (*n*=10, **p*<0.001 sex; •*p*<0.05 *vs.* 10w old mice). **C.** Ambulatory activity of 10w, 20w and 30w old male and female during 16hs of fasting.
