# Supplementary material for: The feeding microstructure of male and female mice
Source: PLoS One. 2021 Feb 4;16(2):e0246569. doi: 10.1371/journal.pone.0246569 (PMC7861458; doi:10.1371/journal.pone.0246569)
Supplement: S5 Fig — A-B. Shown are the mean first nocturnal meal size (A, kCal) and duration (B, min) of group-housed male (n = 10) and female (n = 10) mice at 10w, 20w and 30w of age fed a chow diet ad libitum (blue bars) or after re-feeding following a fasting period of 16hs (red bars). Results represent the mean ± SEM. (PDF) [file pone.0246569.s005.pdf]

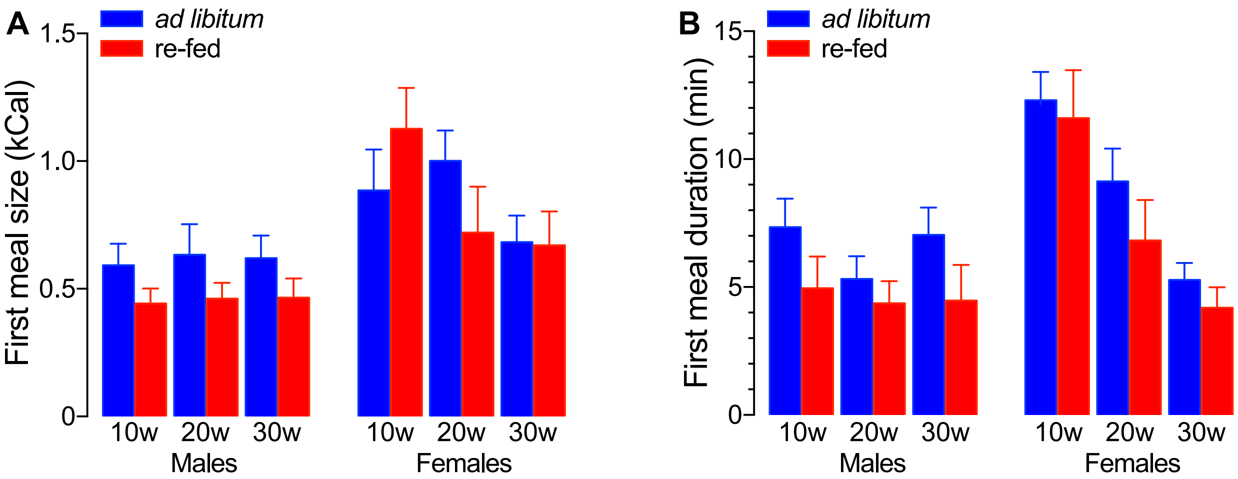

S5 Fig. *First meal size and duration of mice fed ad libitum or after re-feeding.* A-B. Shown are the mean first nocturnal meal size (A, kCal) and duration (B, min) of group-housed male (*n*=10) and female (*n*=10) mice at 10w, 20w and 30w of age fed a chow diet *ad libitum* (blue bars) or after re-feeding following a fasting period of 16hs (red bars). Results represent the mean ± SEM.
