# Supplementary material for: The feeding microstructure of male and female mice
Source: PLoS One. 2021 Feb 4;16(2):e0246569. doi: 10.1371/journal.pone.0246569 (PMC7861458; doi:10.1371/journal.pone.0246569)
Supplement: Attachment — Submitted filename: Responses to the Reviewers v2.docx [file pone.0246569.s007.docx]

**Responses to comments of Reviewer #1**

1. *"The abstract could be more specific and discuss what the sex and age effects were, similar to how they specifically described the BW results in the prior sentences. Similarly, in the next sentence, it would be informative to specifically describe the sex-related decrease in the non-feeding time between meals.”*

Response: We have re-written a section of the abstract to indicate the sex- and age-dependent effects we have found in the feeding microstructure of mice.

2. *"The additional description of the experimental design (e.g., scrambling and group-housing etc.) is helpful to visualize the experiment. It would be even more helpful to add a supplemental figure that shows this experimental design timeline."*

Response: Following the Reviewer's suggestion, a figure reflecting the experimental design time-line is now included as Supplementary Figure 1.

3. *"Regarding the small sample size, the authors have added one phrase to point out the small sample size in the discussion of the lack of correlation between energy expenditure and activity. However, this limitation should be discussed with regard to the entire experiment. The small sample size will affect not only affect activity-energy expenditure relationships, but also the other parameters in the experiment and this should be discussed."*

Response: The Discussion now includes several notes of caution when interpreting our results due to the limitations imposed by a small sample size. They can be found in lines 543, 579 and 632.
